# Identification of a Genotype IX Newcastle Disease Virus in a Guangxi White Duck

**DOI:** 10.1128/genomeA.00836-13

**Published:** 2013-10-10

**Authors:** Zhixun Xie, Liji Xie, Zongli Xu, Jiabo Liu, Yaoshan Pang, Xianwen Deng, Zhiqin Xie, Qing Fan, Sisi Luo

**Affiliations:** Guangxi Key Laboratory of Animal Vaccines and Diagnostics, Guangxi Veterinary Research Institute, Nanning, Guangxi Province, China

## Abstract

We report the complete genomic sequence of a novel Newcastle disease virus (NDV) strain, duck/China/Guangxi19/2011, isolated from a white duck in Guangxi Province, southern China. Phylogenetic analysis based on a fusion gene comparison with different NDV strains revealed that duck/China/Guangxi19/2011 is phylogenetically close to genotype IX NDV, and the deduced amino acid sequence of the fusion protein cleavage site was 112R-R-Q-R-R-F117. The whole nucleotide sequence had the highest homology (99.7%) to the sequence of strain F48E8 (GenBank accession number FJ436302). This study will help us understand the epidemiology and molecular characteristics of genotype IX Newcastle disease virus in ducks.

## GENOME ANNOUNCEMENT

Newcastle disease virus (NDV) is a single-stranded, negative-strand RNA virus. The full genome of NDV has three sequence types, with lengths of 15,198 nucleotides, 15,192 nucleotides, and 15,186 nucleotides. NDVs of 15,198 nucleotides belong to class I (containing 9 genotypes), and NDVs of 15,192 nucleotides and 15,186 nucleotides belong to class II (containing 11 genotypes) (1–3). The NDV genome sequence contains six open reading frames, which encode 6 kinds of proteins (3′-NP-P-M-F-HN-L-5′), nucleocapsid proteins, phosphoproteins, matrix proteins, fusion proteins, hemagglutinin-neuraminidase proteins, and large polymerases (4–6).

In December 2011, NDV was isolated from a white duck in Guangxi Province, southern China. The isolate was named duck/China/Guangxi19/2011. Nucleotide sequences of duck/China/Guangxi19/2011 were amplified by PCR. The amplified products were purified and cloned into the pMD18-T vector (TaKaRa) and then sequenced (TaKaRa, Dalian, China). Sequences were assembled and manually edited to generate the final genome sequence.

Sequence analysis showed that the full genome sequence of duck/China/Guangxi19/2011 is 15,192 nucleotides and has the highest homology (99.7%) to the sequence of strain F48E8 (GenBank accession number FJ436302, class II, genotype IX). The amino acid sequence identities of the NP, P, M, F, HN, and L proteins between duck/China/Guangxi19/2011 and F48E8 are 99.6%, 99.0%, 98.6%, 99.3%, 99.1%, and 99.6%, respectively. The amino acid sequence identities of the NP, P, M, F, HN, and L proteins between duck/China/Guangxi/2011 and strain LaSota (GenBank accession number AF077761, class II, genotype II) are 91.5%, 86.5%, 90.2%, 91.9%, 90.6%, and 93.4%, respectively. The amino acid sequence identities of the NP, P, M, F, HN, and L proteins between duck/China/Guangxi/2011 and the Newcastle disease virus isolate SDWF02 (GenBank accession number HM188399, class II, genotype VII) are 94.5%, 80.8%, 89.2%, 90.3%, 88.3%, and 93.6%, respectively.

The sequence at the fusion protein cleavage site is a major determinant of NDV pathogenicity (7–9). The F gene of duck/China/Guangxi19/2011 has the highest sequence homology (99.8%) to strain F48E8, and its virulence fusion protein cleavage site sequence (112R–R-Q-R-R-F117) (10) is in accord with the detected biological characteristics (mean death time, 51.4 h; intracerebral pathogenicity index, 1.80; intravenous pathogenicity index, 2.82).

The first to 21st amino acid sites of the F protein are the signal peptide areas of the N terminus and comprise one of the main variant areas of the F protein (11). There are four amino acid mutations in duck/China/Guangxi19/2011 compared with strain F48E8. The sites of amino acid mutations are the third amino acid, for which proline (hydrophobic) in F48E8 is mutated to serine (hydrophilic) in duck/China/Guangxi19/2011; the fourth amino acid, for which lysine (alkaline) in F48E8 is mutated to arginine (basic) in duck/China/Guangxi19/2011; the 380th amino acid, for which threonine (hydrophilic) is mutated to alanine (hydrophobic) in duck/China/Guangxi19/2011; and the 553rd amino acid, for which methionine (hydrophobic) is mutated to isoleucine (hydrophobic) in duck/China/Guangxi19/2011. Further study is needed to determine whether these variations affect viral fusion.

This report of the phylogenetic analysis of the whole-genome sequence of genotype IX NDV isolated from a white duck will further understanding of the epidemiology and molecular characteristics of NDV in duck.

### Nucleotide sequence accession number.

The GenBank accession number for duck/China/Guangxi19/2011 is KC920893.
